# Seroprevalence of vector-borne pathogens in outdoor workers from southern Italy and associated occupational risk factors

**DOI:** 10.1186/s13071-022-05385-6

**Published:** 2022-07-25

**Authors:** Angela Stufano, Roberta Iatta, Giovanni Sgroi, Hamid Reza Jahantigh, Francesco Cagnazzo, Agnes Flöel, Guglielmo Lucchese, Daniela Loconsole, Francesca Centrone, Jairo Alfonso Mendoza-Roldan, Maria Chironna, Domenico Otranto, Piero Lovreglio

**Affiliations:** 1 Interdisciplinary Department of Medicine, University of Bari, Bari, Italy; 2 Department of Veterinary Medicine, University of Bari, Valenzano, Italy; 3 Department of Neurology, University Medicine Greifswald, Greifswald, Germany; 4 German Center for Neurodegenerative Diseases Rostock-Greifswald, Greifswald, Germany; 5 Faculty of Veterinary Sciences, Bu-Ali Sina University, Hamedan, Iran

**Keywords:** Farmers, Chemiluminescent immunoassay, Tick borne pathogens, *Coxiella burnetii*, *Rickettsia conorii*, Zoonosis, Public health

## Abstract

**Background:**

Vector-borne diseases (VBDs) represent an emerging global threat to public health due to the geographical expansion of arthropod vectors. The study aims to assess the seroprevalence of selected vector-borne pathogens (VBPs) in different groups of outdoor workers and the occupational risk factors for exposure to arthropod bites.

**Methods:**

A cross-sectional study was conducted on 170 workers recruited in two different regions of southern Italy, including farmers, forestry workers, veterinarians, geologists/agronomists and administrative employees, and tested for IgG antibodies against *Bartonella henselae*, *Borrelia* spp. *Coxiella burnetii* and *Rickettsia conorii*, using a chemiluminescent immunoassay (CLIA). The relationship among job characteristics, tick exposure and the prevalence of seropositive subjects for each pathogen was investigated by applying categorical principal component analysis (CATPCA).

**Results:**

A high seroprevalence for *C. burnetii* (30.0%) and *R. conorii* (15.3%) was reported, mainly in farmers (67.7% and 54.8%, respectively) and forestry workers (29.0% and 16.1%, respectively), while a low prevalence was observed for *B. henselae* and *Borrelia* spp. (8.8% and 4.1%, respectively). The regression equation by CATPCA was significant for *C. burnetii* and *R. conorii* (*P* < 0.001), showing a positive association with job, tick bite exposure, working area and contact with animals.

**Conclusions:**

These findings highlight the need of activating an appropriate occupational health response for minimizing the risk of arthropod vector exposure in workplaces, considering specific preventive measures in particular in high-risk job categories.

**Graphical Abstract:**

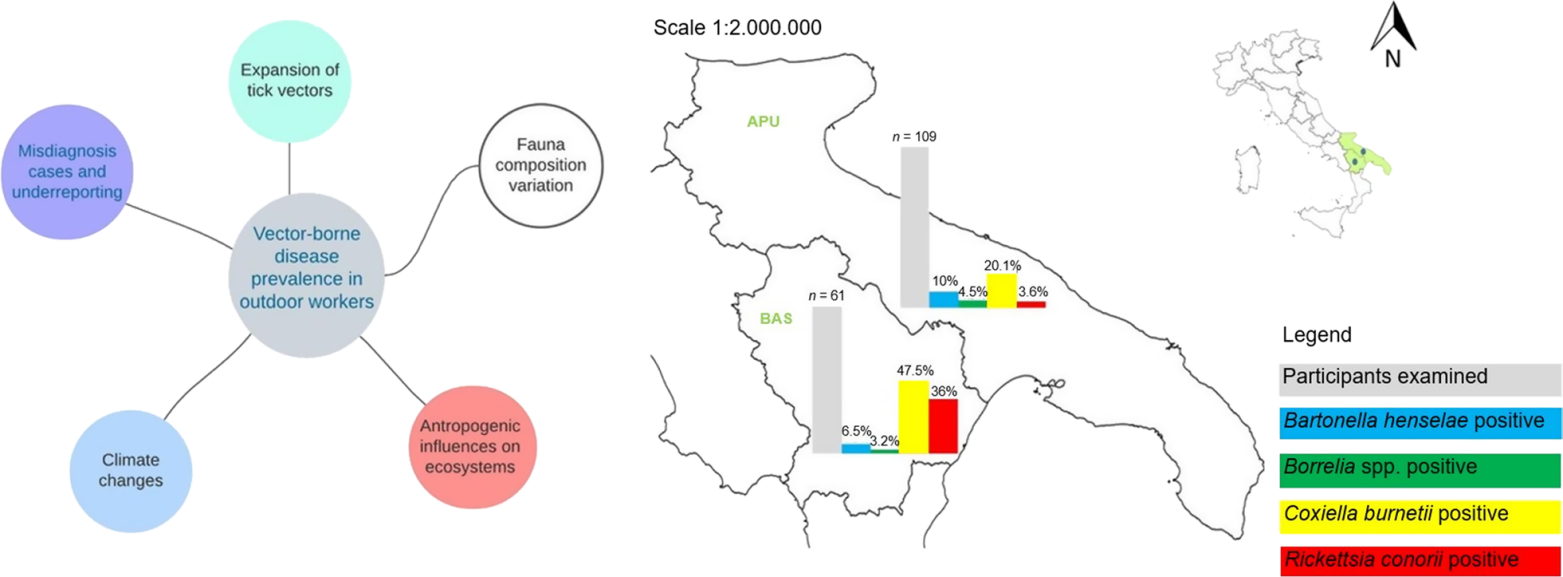

## Background

Vector-borne diseases (VBD) are increasingly threating animal and human health worldwide, being caused by a wide range of pathogens transmitted by arthropod vectors, including ticks. Among them, Lyme disease, tick-borne encephalitis, Q fever, bartonellosis and numerous tick-borne rickettsioses are expanding in previous non-endemic areas, overlapping the distribution of the vectors [1–3]. In particular, *Ixodes ricinus*, the wood tick, is largely distributed in Europe and could transmit various pathogens, including *Borrelia burgdorferi* sensu lato complex and *Rickettsia* species of the spotted fever group [4–6]. The brown dog tick, *Rhipicephalus sanguineus* (s.l.), also has a large distribution in Europe, with high frequency in the Mediterranean regions being usually involved in the transmission of *Rickettsia conorii* [7]. In addition, *Dermacentor marginatus* and *R. sanguineus* (s.l.) may shed in faeces *Coxiella burnetii*, causing Q fever [8]. Furthermore, a high circulation of *I. ricinus*, *D. marginatus* and *R. sanguineus* (s.l.) ticks and related zoonotic pathogens was recorded in synanthropic animals and humans in the southern regions of Italy [9–11].

Overall, reports on tick-borne diseases (TBDs) are increasing because of the alarming geographical expansion of tick vectors, especially in new geographic areas of the northern hemisphere, leading to an increased public health burden [12] as a consequence of multifactorial reasons such as animal movement [13], anthropogenic influence on many ecosystems, variation in vertebrate fauna composition and recreational social changes [14–16]. Though the incidence of TBDs in humans is still underestimated because of the low number of case notifications and the frequency of atypical onset of clinical manifestations, TBDs are gaining the interest of the scientific community [3] because they may lead to chronic forms, severe illness and death, depending on the balance between host immune system and pathogenic factors [16]. Moreover, few studies have focused on the seroprevalence rate of TBPs in exposed populations, particularly, little is known about specific occupational risk factors [17, 18]. Previous researches found that individuals employed in land and animal management activities are at risk of exposure to tick bite and TBDs, and outdoor workers were approximately 3–10 times more likely to be infected by TBPs [19, 20]. Nonetheless, no data are available on specific categories, such as geologists and agronomists, and only few occupational studies [21, 22] have been conducted in at-risk workers to simultaneously assess the prevalence of different TBPs alone or in combination. Therefore, this study was carried out to detect the exposure of outdoor workers to TBPs and to determine the job characteristics and the occupational factors that pose a higher risk of TBP infection.

## Methods

### Study population

The cross-sectional study was conducted in the period February–September 2021 on 170 workers performing different jobs including outdoor activities, namely forestry workers, farmers, veterinarians, geologists and agronomists (Table 1). The forestry workers and the farmers participated in the study as volunteers during educational meetings, whereas veterinarians, geologists and agronomists were recruited among the employees of the University of Bari at the time of the occupational health surveillance. Geologists and agronomists were considered as a single group according to the similar tasks related to the potential tick exposure (outdoor activity with limited occupational contact with animals). A control group of administrative university employees, not performing occupational tasks involving animal contact or outdoor activities, was also voluntary recruited. All the participants had to fulfil the following inclusion criteria: being older than 18 years and no prior history of immunodeficiency.

**Table 1:** General and occupational characteristics according to the questionnaires in the studied population based on job category

Job category	Age (years) median (range)	Working seniority (years) median (range)	Female gender (%)^a^	Potential occupational tick exposure (%)^a^	Tick bites at work: lifetime/previous year (%)^a^	Ticks on clothes at work: lifetime/previous year (%)^a^	Work area: lowland/mountain (%)^a^	Work area: wetland (%)^a^	Occupational contact with animals (%)^a^
Forestry workers (*n* = 31)	41.0 (26–68)	10.0 (2–35)	32.2	100.0	80.7/41.9	74.2/54.5	0.0/83.8	93.5	25.0
Farmers (*n* = 31)	46.5 (23–71)	15.0 (1–30)	9.3	100.0	100.0/74.1	90.3/77.5	6.4/96.7	96.7	93.5
Veterinarians (*n* = 44)	42.5 (25–69)	11.0 (1–30)	40.9	86.4	27.3/6.8	34.1/18.8	31.8/11.3	40.9	100.0
Geologists/Agronomists (*n* = 30)	53.0 (26–64)	20.0 (1–32)	30.0	86.7	36.7/10.0	40.0/23.3	40.0/43.3	73.3	10.0
Administrative employees (*n* = 34)	49.5 (27–66)	15.0 (1–32)	67.6	0.0	0.0/0.0	0.0/0.0	73.5/0.0	0.0	0.0
Total (*n* = 170)	47.0 (23–71)	14.5(1–35)	37.1	78.2	47.0/23.5	46.4/37.0	32.2/46.5	58.0	49.4

^a^*P* < 0.001

All the participants filled in a standardized questionnaire enquiring about socio-demographic and job characteristics, previous and potential exposure to ticks in the work environment and during leisure time, and potential clinical history of TBDs.

### Study areas

**Fig. 1:**
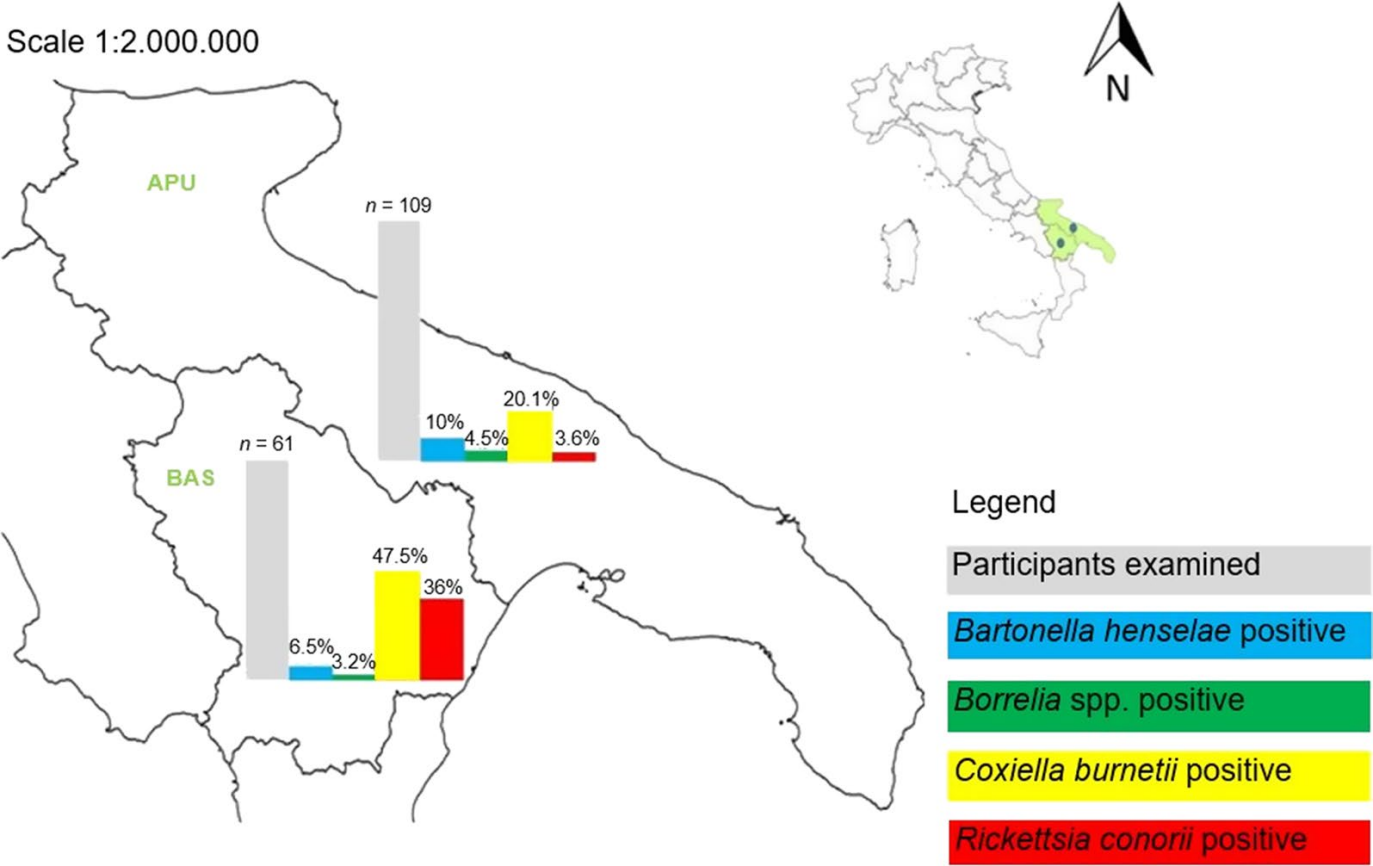
TBP seroprevalence rates of recruited workers in Apulia (APU) and Basilicata (BAS) regions

Workers were recruited in two different areas of southern Italy: forestry workers and farmers from the Parco Regionale di Gallipoli Cognato-Piccole Dolomiti Lucane, in the Basilicata region, whereas the university employees were from the province of Bari in the Apulia region (Fig. 1). The areas were chosen based on previous studies on the occurrence and seasonality of questing ticks from the environment [6, 23–25] and on the detection and seroprevalence of several vector-borne pathogens in synanthropic mammals and in exposed workers [9, 25, 26]. The two areas are characterized by a typical Mediterranean temperate climate with progressive continental features in inland and mountainous landscapes, with hot and dry summer and moderately cold and rainy winter season [27].

### Serological examination

For each enrolled worker, a blood sample (10 ml) was collected in a Vacutainer tube. Serum samples, obtained after centrifugation at 2000×*g* for 10 min, were stored at −20 °C until the analysis. Serum samples were tested for IgG antibodies anti-*B*. *henselae, Borrelia* spp., *C*. *burnetii* and *R*. *conorii* using a chemiluminescent immunoassay (CLIA, Vircell^®^, S.L.). The results of CLIA were expressed using the antibody index calculated as the ratio between the sample Relative Light Unit (RLU) and the calibrator RLU. The VirClia^®^ IgG assay showed sensitivity ranging from 79% to 95.9% and specificity from 93.8% to 97.9% on *C. burnetii*, *B. henselae* and *Borrelia* spp., agreeing when compared with both ELISA and IFA testing [28–30]. Results were interpreted according to the manufacturer’s instructions. Briefly, each assay consists of three reaction wells and five reagent wells (namely the conjugate containing anti-human IgG antibodies coupled with peroxidase, the serum dilution solution, the calibrator and two substrate components (namely peroxide and luminol). The samples are diluted at 1:20, and the results are expressed as an “antibody index (AI)” (= sample relative light unit (RLU)/calibrator RLU), where samples with indexes < 0.9 are considered negative, while samples > 1.1 are considered positive. Samples with an index between both values were considered equivocal and were retested.

All samples were analysed at the Laboratory of Molecular Epidemiology and Public Health, Department of Biomedical Science and Human Oncology of the University of Bari, Italy.

### Statistical analysis

The analyses were performed with SPSS 28 (IBM Corp., Armonk, NY, USA). Continuous variables are described as median and range, while categorical variables as raw frequency and percentage. The relationship between general and job characteristics, tick exposure and previous tick bites and the prevalence of seropositive subjects for each pathogen were investigated by applying dimensionality reduction followed by linear regression. Non-linear principal component analysis (NLPCA), also known as categorical principal component analysis (CATPCA), was used to map the variables onto a lower dimensional space. The CATPCA allows to extend linear PCA to ordinal and nominal categorical variables while exploring possible non-linear relationships [31–33]. Age and working seniority were considered numeric variables and discretized by multiplication; all other variables were quantified as nominal. A set of five-dimension solutions or components was identified by linearly combining the original correlated variables in an appropriate manner using non-parametric bootstrap to assess significance of the loadings on the components. Eight-, seven-, six- and five-dimension solutions were computed. The five-dimension solution was adopted for analysis since all the confidence intervals of the loadings on the last component within the other solutions included the value zero while the first five components explained ≈42% of the total variance indicating adequate fit [33]. Variables loading with coefficient absolute values ≥ ± 0.4 were considered to have a significant effect on the component [33]. Finally, we performed linear regression using each of the five series of CATPCA scores, one for each component, as predictors for seropositivity for each pathogen [33]. The significance threshold for regression analysis was set at 0.007 after Bonferroni correction, whereas a *P*-value < 0.05 was considered statistically significant for all the analyses.

## Results

Data regarding general and occupational characteristics of the studied population subdivided according to job are reported in Table 1. No statistically significant difference among the worker groups was observed according to the age and working seniority. Potential occupational exposure to ticks was statistically higher in farmers and forestry workers (100.0%), who also reported significantly higher experience of tick bites (100.0% and 80.7%, respectively), detection of ticks on clothes during work activities (90.3% and 74.2%, respectively) and frequency of working in wet areas (96.7% and 93.5%, respectively) (always *P* < 0.001).

An overall seroprevalence of 45.2% (77/170) for at least one TBP was recorded in workers enrolled. The seropositivity for each TBP investigated, for at least one TBP and for more than one TBP for each job category, is reported in Table 2. A significantly higher seroprevalence was observed for *C. burnetii* and *R. conorii* in farmers than in administrative employees (*P* < 0.001), while no significant differences were found among the workers for *Borrelia* spp. and *B. henselae*. The percentage of subjects with IgG for at least one TBP and/or multiple TBPs was significantly higher in the group of farmers (*P* < 0.001). The main multiple seropositivities were to *C. burnetii* and *R. conorii* (*n* = 15), followed by four cases of seropositivity for more than one TBP by *B. henselae* and *C. burnetii,* and one case of coinfection by *C. burnetii*, *Borrelia* spp. and *R. conorii* (data not shown). The seroprevalence rate for the TBPs recorded in both southern regions (Fig. 1) is significantly higher for *R. conorii* and *C. burnetii* than for other pathogens (*P* < 0.001).

**Table 2:** Seroprevalence (%) of the studied population to each TBP investigated and to multiple TBPs according to job category

Job category	*Bartonella henselae*	*Borrelia* spp.	*Coxiella burnetii*^a^	*Rickettsia conorii*^a^	Seropositivity for at least one TBPs^a^	Seropositivity for more than one TBPs^a^
Forestry workers (*n* = 31)	6.5%	6.5%	29.0%	16.1%	45.1%	12.9%
Farmers (*n* = 31)	6.5%	0.0%	67.7%	54.8%	83.8%	41.9%
Veterinarians (*n* = 44)	6.8%	6.8%	18.2%	4.5%	31.8%	2.2%
Geologists/agronomists (*n* = 30)	10.0%	0.0%	26.7%	6.7%	33.3%	6.6%
Administrative employees (*n* = 34)	14.7%	5.9%	14.7%	0.0%	35.2%	2.9%
Total sample (*n* = 170)	8.8%	4.1%	30.0%	15.3%	45.2%	12.3%

^a^*P* < 0.001

**Fig. 2:**
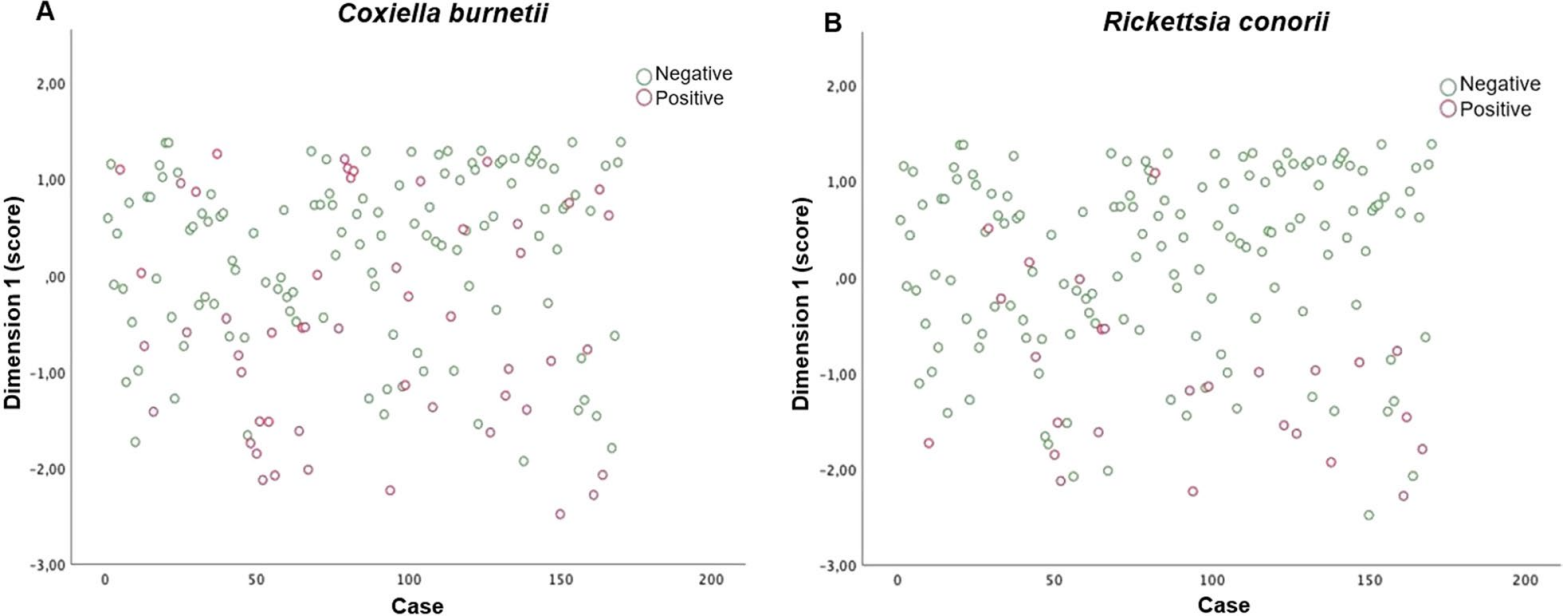
Relationship between the first dimension identified by CATPCA and the seroconversion of the workers for *Coxiella burnetii* (**A**) and *Rickettsia conorii* (**B**)

**Fig. 3:**
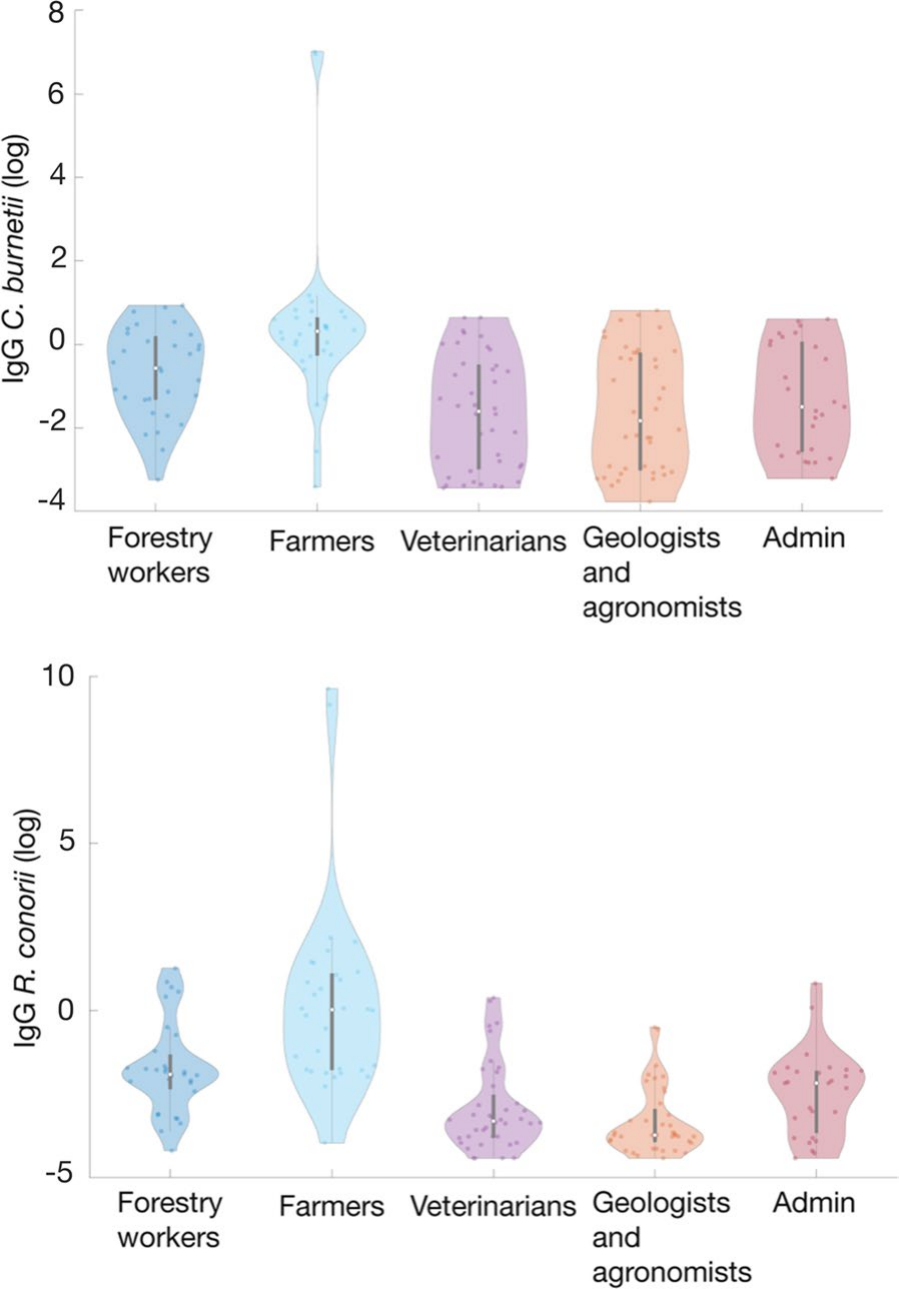
Seropositivity for *Coxiella burnetii* and *Rickettsia conorii* expressed as a function of job. The violin plot shows the median, the values between the 2nd and 3rd quartile and kernel density estimates

Logistic regression was performed using the five components to which general and occupational characteristics were reported as predictors of numbers of seropositive workers for a single TBP investigated. A significant regression equation was found only with the first component for the seropositivity for *C. burnetii* (*B* = −0.734, *P* < 0.001, model omnibus test *P* < 0.001, Nagelkerke-*R*^2^ = 0.365) and *R. conorii* (*B* = −1.218, *P* < 0.001, model omnibus test *P* < 0.001, Nagelkerke-*R*^2^ = 0.243). The first rotated component, therefore, negatively predicted the seropositivity for both *C. burnetii* and *R. conorii* (Fig. 2). The job and a cluster of variables related to the working tasks and the occupational tick exposure loaded with a coefficient higher than ± 0.4, contributing the most to the first component, which accounted for 19% of the variance in the data (Table 3). All the variables mainly contributing to the first component were considered as nominal in the CATPCA; therefore, their relationship with the first component cannot be assumed to be linear. Job, work area, working time at 3–6 p.m., tick exposure and bite, and contact with animals were among the variables contributing most to the first rotated dimension (Table 3). In detail, the job was one of the main contributing variables with a loading > 0.8, indicating a significant association of the occupation type with the seropositivity for *C. burnetii* and *R. conorii*, with farmers presenting higher antibody titers against the two pathogens (Fig. 3). Seronegative cases for both pathogens clustered around higher score values and vice versa for seropositive cases, in accordance with the results of the logistic regression indicating that score values were a significant predictor of serological status.

**Table 3:** Variables loading on the first dimension (variance accounted for 19%) with absolute values ≥  ± 0.4

Variable	Loading
Job	0.845
Working time	
3–6 p.m.	0.534
Work area	
Lowland	0.486
Mountain	−0.676
Wetland	−0.596
Tick exposure	
Bites in lifetime	−0.903
Bite site	−0.854
Local reaction	−0.800
On clothes in working hours	−0.791
Working exposure	−0.630
Working contact with animals	
Cattle	−0.664
Sheep	−0.629
Poultry	−0.559
Swine	−0.538
Horses	−0.439
Milking activity	−0.620
Delivery assistance	−0.552
Number of livestock bred	−0.628

## Discussion

A high seroprevalence of *C. burnetii* (30%) and *R. conorii* (15.3%) was detected in farmers and other outdoor workers, suggesting a potential increased risk for VBDs also related to the high occupational risk of tick exposure. These percentages agree with the tick bite exposure during working activities reported by farmers (i.e. 100%) and forestry workers (i.e. 80.7%), agronomists/geologists (i.e. 36.7%) and veterinarians (i.e. 27.7%). Similar percentages of tick exposure are reported in farmers from Germany (73.6%) [34] and Poland (87.0%) [35] and in forestry workers from Belgium (94.8%) [36]. However, although ticks display an anthropophilic feeding behaviour in absence of their proper host, the high percentage of tick bites reported by farmers and forestry workers is also be likely due to the scarce adherence to preventive measures and protective habits of these workers [37].

The high prevalence of *C. burnetii* exposure in farmers (67.7%) suggests the occurrence of a high risk of infection in the farming environment due to the contact with contaminated aerosols or infected animal products such as placentas [38, 39]. In addition, this seroprevalence higher than that reported in other Italian areas (e.g. 62.9% in Sicily, 50.0% in northern Italy), may suggest a potential wide circulation of ticks infected by *C. burnetii* in the investigated area [25, 40, 41]. This finding was confirmed by the seroprevalence of *C. burnetii* also in forestry workers (29.0%), much higher than in previous studies from northeastern Italy (2.8%), The Netherlands (6.4%), Poland (6.4%) and Germany (6.0%) [22, 42–44]. The seroprevalence of *C. burnetii* (26.7%) recorded in geologists and agronomists suggests that this infection may have a work-related character due to the ubiquitous presence of the bacterium in the rural and wild environments, irrespective of the contact with animals, which was reported by only 10% of these workers. The *C. burnetii* exposure in university veterinary workers (18.2%) is similar to that reported in a previous survey performed in a non-vaccinated veterinary population [45–47] confirming the increased risk in these workers compared to general population.

Finally, also *C. burnetii* prevalence rate in our control group (i.e. administrative workers) (14.7%) was higher than that reported for the general adult population, without specific risk factors, from Europe and the US [41, 48]. This finding could be explained considering that Q fever cases are often underdiagnosed because of its non-specific symptoms, often related to the virulence of the involved strain and to the host adaptation [49].

To date, only two reports evaluated *R. conorii* in workers from Italy [25, 44] showing a seroprevalence rate of 3.9% in a Northern Italian area and 5.0% in a population of forestry workers from the same area of our study [25], considerably lower than that in the farmers (54.8%) and forestry workers (16.1%) herein investigated, with the latter similar to that reported in France, Poland and Germany ranging from 9.2 to 27.0% [50–52].

Although the seroprevalence rates for *B. henselae* was higher in administrative employees (i.e. 14.7%), no significant differences were reported among worker groups, being similar to that reported in the Italian general adult population, ranging from 6.3 to 13.0% [53–55], thus representing a minor risk for job categories listed here.

Differently from reports of *Borrelia burdorferi* exposure in high-risk workers from different Italian regions [37–41] and Europe [22, 36], a low seroprevalence (4.1%) was herein observed, which is in accordance with the absence of clinical cases of Lyme disease in this geographical area. The multiple pathogen exposure (i.e. 12.3%), higher than that recorded in outdoor workers from highly endemic regions ranging from 4.7% to 7.6% in previous studies [56, 57], suggests the worker exposure to a single co-infected tick or to multiple ticks.

The CAPTCA analysis showed that the seroprevalence for *C. burnetii* and *R. conorii* is positively associated with three major groups of variables (i.e. tick exposure, working environment and occupational contact with animals). Particularly, high coefficients have been observed not only for variable such as previous tick bites and finding ticks on clothes during working hours, but also for those related to the site and the local clinical reaction related to a tick bite.

While it is known that ticks are vectors of *Rickettsia* spp., their impact on the epidemiology of *C. burnetii* infection is still to be defined because of the role of alternative routes of transmission [38]. Although this pathogen has been detected in ticks, the infection in livestock or the forestry context through tick bites or inhalation of aerosol contaminated by *C. burnetii* is still unknown [58]. Regression analysis has also shown that contact with livestock animals (e.g. cattle, sheep and goats) represents a major occupational risk factor for acquiring *C. burnetii* infection, as previously described by two Q fever outbreaks in Italian farmers exposed to infected sheep [59, 60].

Working in wetlands and mountain areas is significantly associated with a higher seroprevalence of *C. burnetii* and *R. conorii* as altitude is a determinant factor for the presence of tick species as demonstrated for *I. ricinus* in southern Italy, being collected at high altitude levels (> 1000 m) during all seasons [23]. Moreover, wooded areas are characterized by a microclimate with variable temperatures, low wind speed and high moisture, while an open landscape is less favourable for ticks because of their low desiccation resistance [61].

However, antibody reduction over time and heterogeneity in individual antibody response may affect this kind of seroepidemiological survey along with the occurrence of cross-reactivity reactions in the identification of different species of pathogens belonging to the same genus.

Nonetheless, the high seroprevalence of farmers and forestry workers to *C. burnetii* and *R. conorii* suggests an occupational risk for these job categories in an area where the tick fauna is one of the most diverse across Europe [23].

## Conclusion

Overall, these data may spur the interest in confirming and extending seroprevalence studies in broader occupational exposed populations for better evaluating the clinical implications of these TBDs. Moreover, use of a single-assay chemiluminescent test system, which is very simple to perform and requires minimum sample handling, eliminates the need to make serum dilutions at high concentrations, avoids variations related to the conventional manual or semiautomatic techniques such as ELISA and seems to be a highly advantageous option for seroprevalence studies especially in occupational settings [62]. It could not be ruled out that cases of human TBDs may remain underdiagnosed because of the non-specific disease presentation and lack of awareness of physicians about their diagnosis. All these factors should be considered in the epidemiology of TBDs, being pieces of the puzzle that required the activation of an appropriate public and occupational health response for minimizing the risk in workplaces including vaccination promotion against Q fever in high-risk job categories.

## Data Availability

All data and materials in the present work are available upon request to the correspondent Author.

## References

[1] Heyman P, Cochez C, Hofhuis A, van der Giessen J, Sprong H, Porter SR (2010). A clear and present danger: tick-borne diseases in Europe. Expert Rev Anti Infect Ther.

[2] Kilpatrick AM, Randolph SE (2012). Drivers, dynamics, and control of emerging vector-borne zoonotic diseases. Lancet.

[3] Dantas-Torres F, Otranto D (2016). Best practices for preventing vector-borne diseases in dogs and humans. Trends Parasitol.

[4] Stanek G, Wormser GP, Gray J, Strle F (2012). Lyme borreliosis. Lancet.

[5] Dantas-Torres F, Chomel BB, Otranto D (2012). Ticks and tick-borne diseases: a One Health perspective. Trends Parasitol.

[6] Lorusso V, Lia RP, Dantas-Torres F, Mallia E, Ravagnan S, Capelli G (2011). Ixodid ticks of road-killed wildlife species in southern Italy: new tick-host associations and locality records. Exp Appl Acarol.

[7] Dantas-Torres F (2008). The brown dog tick, *Rhipicephalus sanguineus* (Latreille, 1806) (Acari: Ixodidae): from taxonomy to control. Vet Parasitol.

[8] Maurin M, Raoult D (1999). Q fever. Clin Microbiol Rev.

[9] Sgroi G, Iatta R, Lia RP, Napoli E, Buono F, Bezerra-Santos MA (2021). Tick exposure and risk of tick-borne pathogens infection in hunters and hunting dogs: a citizen science approach. Transbound Emerg Dis.

[10] Sgroi G, Iatta R, Veneziano V, Bezerra-Santos MA, Lesiczka P, Hrazdilová K (2021). Molecular survey on tick-borne pathogens and *Leishmania infantum* in red foxes (*Vulpes vulpes*) from southern Italy. Ticks Tick Borne Dis.

[11] Sgroi G, Iatta R, Lia RP, D'Alessio N, Manoj RRS, Veneziano V (2021). Spotted fever group rickettsiae in *Dermacentor marginatus* from wild boars in Italy. Transbound Emerg Dis.

[12] Otranto D, Dantas-Torres F (2010). Canine and feline vector-borne diseases in Italy: current situation and perspectives. Parasit Vectors.

[13] Rizzoli A, Silaghi C, Obiegala A, Rudolf I, Hubálek Z, Földvári G (2014). *Ixodes ricinus* and its transmitted pathogens in urban and peri-urban areas in Europe: new hazards and relevance for Public Health. Front Public Health.

[14] Otranto D, Cantacessi C, Pfeffer M, Dantas-Torres F, Brianti E, Deplazes P (2015). The role of wild canids and felids in spreading parasites to dogs and cats in Europe. Part I: protozoa and tick-borne agents. Vet Parasitol.

[15] Soares TCB, Isaias GAB, Almeida AR, Drummond MR, da Silva MN, Lania BG (2020). Prevalence of *Bartonella* spp. infection in patients with sickle cell disease. Vector Borne Zoonotic Dis.

[16] Otranto D, Dantas-Torres F, Giannelli A, Latrofa MS, Cascio A, Cazzin S (2014). Ticks infesting humans in Italy and associated pathogens. Parasit Vectors.

[17] Tomao P, Ciceroni L, D'Ovidio MC, De Rosa M, Vonesch N, Iavicoli S (2005). Prevalence and incidence of antibodies to *Borrelia burgdorferi* and to tick-borne encephalitis virus in agricultural and forestry workers from Tuscany, Italy. Eur J Clin Microbiol Infect Dis.

[18] Piacentino JD, Schwartz BS (2002). Occupational risk of Lyme disease: an epidemiological review. Occup Environ Med.

[19] Verso MG, Vesco G, Villari S, Galluzzo P, Gargano V, Matranga D (2016). Analysis of seroprevalence against *Coxiella burnetii* in a sample of farm workers in Western Sicily. Ann Agric Environ Med.

[20] Jurke A, Bannert N, Brehm K, Fingerle V, Kempf VA, Kömpf D (2015). Serological survey of *Bartonella* spp., *Borrelia burgdorferi*, Brucella spp., *Coxiella burnetii*, *Francisella tularensis*, *Leptospira* spp., *Echinococcus*, Hanta-, TBE- and XMR-virus infection in employees of two forestry enterprises in North Rhine-Westphalia, Germany, 2011-2013. Int J Med Microbiol.

[21] Chmielewska-Badora J, Moniuszko A, Żukiewicz-Sobczak W, Zwoliński J, Piątek J, Pancewicz S (2012). Serological survey in persons occupationally exposed to tick-borne pathogens in cases of co-infections with *Borrelia burgdorferi*, *Anaplasma phagocytophilum*, *Bartonella* spp. and *Babesia microti*. Ann Agric Environ Med.

[22] Rigaud E, Jaulhac B, Garcia-Bonnet N, Hunfeld KP, Féménia F, Huet D (2016). Seroprevalence of seven pathogens transmitted by the *Ixodes ricinus* tick in forestry workers in France. Clin Microbiol Infect.

[23] Dantas-Torres F, Otranto D (2013). Species diversity and abundance of ticks in three habitats in southern Italy. Ticks Tick Borne Dis.

[24] Falchi A, Dantas-Torres F, Lorusso V, Malia E, Lia RP, Otranto D (2012). Autochthonous and migratory birds as a dispersion source for *Ixodes ricinus* in southern Italy. Exp Appl Acarol.

[25] Mendoza-Roldan JA, Ravindran Santhakumari Manoj R, Latrofa MS, Iatta R, Annoscia G, Lovreglio P (2021). Role of reptiles and associated arthropods in the epidemiology of rickettsioses: a one health paradigm. PLoS Negl Trop Dis.

[26] Bezerra-Santos MA, Sgroi G, Mendoza-Roldan JA, Khedri J, Camarda A, Iatta R (2020). Ectoparasites of hedgehogs: from flea mite phoresy to their role as vectors of pathogens. Int J Parasitol Parasites Wildl.

[27] Rundel PW (1998). Landscape disturbance in Mediterranean-type ecosystems: an overview. Landscape disturbance and biodiversity in Mediterranean-type ecosystems. Ecol Stud.

[28] Bayart JL, Gusbin C, Lardinois B, Scohy A, Kabamba-Mukadi B (2020). Analytical and clinical evaluation of new automated chemiluminescent immunoassays for the detection of IgG and IgM anti-*Bartonella henselae* antibodies. Diagn Microbiol Infect Dis.

[29] Fernández-Blazquez A, Fernández-Blazquez A, Sabater C, Cuesta-Gonzalez G, Diaz-Carrio MC, Alvarez-Candanedo AR (2018). Comparison of new chemiluminescent immunoassays with indirect immunofluorescence assay in the diagnosis of human Q fever.

[30] Hoeve-Bakker BJA, Jonker M, Brandenburg AH, den Reijer PM, Stelma FF, van Dam AP (2022). The performance of nine commercial serological screening assays for the diagnosis of Lyme borreliosis: a multicenter modified two-gate design study. Microbiol Spectr.

[31] Linting M, van der Kooij A (2012). Nonlinear principal components analysis with CATPCA: a tutorial. J Pers Assess.

[32] Meulman JJ, Van der Kooij AJ, Heiser WJ, Kaplan D (2004). Principal components analysis with nonlinear optimal scaling transformations for ordinal and nominal data. The Sage handbook of quantitative methodology for the social sciences.

[33] Kemalbay G, Korkmazoğlu ÖB (2014). Categorical principal component logistic regression: a case study for housing loan approval. Procedia Soc Behav Sci.

[34] Schielein L, Tizek L, Biedermann T, Zink A (2022). Tick bites in different professions and regions: pooled cross-sectional study in the focus area Bavaria, Germany. BMC Public Health.

[35] Cisak E, Sroka J, Zwoliński J, Umiński J (1998). Seroepidemiologic study on tick-borne encephalitis among forestry workers and farmers from the Lublin region (eastern Poland). Ann Agric Environ Med.

[36] De Keukeleire M, Robert A, Luyasu V, Kabamba B, Vanwambeke SO (2018). Seroprevalence of *Borrelia burgdorferi* in Belgian forestry workers and associated risk factors. Parasit Vectors.

[37] Riccò M, Bragazzi NL, Vezzosi L, Balzarini F, Colucci ME, Veronesi L (2020). Knowledge, attitudes, and practices on tick-borne human diseases and tick-borne encephalitis vaccine among farmers from North-Eastern Italy. J Agromedicine.

[38] Groten T, Kuenzer K, Moog U, Hermann B, Maier K, Boden K (2020). Who is at risk of occupational Q fever: new insights from a multi-profession cross-sectional study. BMJ Open.

[39] Pouquet M, Bareille N, Guatteo R, Moret L, Beaudeau F (2020). *Coxiella burnetii* infection in humans: to what extent do cattle in infected areas free from small ruminants play a role?. Epidemiol Infect.

[40] Fenga C, Gangemi S, De Luca A, Calimeri S, Lo Giudice D, Pugliese M (2015). Seroprevalence and occupational risk survey for *Coxiella burnetii* among exposed workers in Sicily, southern Italy. Int J Occup Med Environ Health.

[41] Tabibi R, Baccalini R, Barassi A, Bonizzi L, Brambilla G, Consonni D (2013). Occupational exposure to zoonotic agents among agricultural workers in Lombardy Region, northern Italy. Ann Agric Environ Med.

[42] Cinco M, Luzzati R, Mascioli M, Floris R, Brouqui P (2006). Serological evidence of *Rickettsia* infections in forestry rangers in north-eastern Italy. Clin Microbiol Infect.

[43] Moll van Charante AW, Groen J, Mulder PG, Rijpkema SG, Osterhaus AD (1998). Occupational risks of zoonotic infections in Dutch forestry workers and muskrat catchers. Eur J Epidemiol.

[44] Żukiewicz-Sobczak W, Zwoliński J, Chmielewska-Badora J, Galińska EM, Cholewa G, Krasowska E (2014). Prevalence of antibodies against selected zoonotic agents in forestry workers from eastern and southern Poland. Ann Agric Environ Med.

[45] Sellens E, Bosward KL, Norris JM, Wood N, Heller J, Graves S (2020). *Coxiella burnetii* seroprevalence in unvaccinated veterinary workers in Australia: evidence to support Q fever vaccination. Zoonoses Public Health.

[46] Whitney EA, Massung RF, Candee AJ, Ailes EC, Myers LM, Patterson NE (2009). Seroepidemiologic and occupational risk survey for *Coxiella burnetii* antibodies among US veterinarians. Clin Infect Dis.

[47] Abe T, Yamaki K, Hayakawa T, Fukuda H, Ito Y, Kume H (2001). A seroepidemiological study of the risks of Q fever infection in Japanese veterinarians. Eur J Epidemiol.

[48] Nielsen SY, Andersen AM, Mølbak K, Hjøllund NH, Kantsø B, Krogfelt KA (2013). No excess risk of adverse pregnancy outcomes among women with serological markers of previous infection with *Coxiella burnetii*: evidence from the Danish National Birth Cohort. BMC Infect Dis.

[49] Sobotta K, Hillarius K, Jiménez PH, Kerner K, Heydel C, Menge C (2018). Interaction of *Coxiella burnetii* strains of different sources and genotypes with bovine and human monocyte-derived macrophages. Front Cell Infect Microbiol.

[50] Fournier PE, Grunnenberger F, Jaulhac B, Gastinger G, Raoult D (2000). Evidence of *Rickettsia helvetic*a infection in humans, eastern France. Emerg Infect Dis.

[51] Podsiadły E, Chmielewski T, Karbowiak G, Kędra E, Tylewska-Wierzbanowska S (2011). The occurrence of spotted fever rickettsioses and other tick-borne infections in forest workers in Poland. Vector Borne Zoonotic Dis.

[52] Wölfel S, Speck S, Essbauer S, Thoma BR, Mertens M, Werdermann S (2017). High seroprevalence for indigenous spotted fever group rickettsiae in forestry workers from the federal state of Brandenburg, Eastern Germany. Ticks Tick Borne Dis.

[53] Mansueto P, Pepe I, Cillari E, Arcoleo F, Micalizzi A, Bonura F (2012). Prevalence of antibodies anti-*Bartonella henselae* in western Sicily: children, blood donors, and cats. J Immunoassay Immunochem.

[54] Picascia A, Pagliuca C, Sommese L, Colicchio R, Casamassimi A, Labonia F (2017). Seroprevalence of *Bartonella henselae* in patients awaiting heart transplant in southern Italy. J Microbiol Immunol Infect.

[55] Del Prete R, Fumarola D, Fumarola L, Basile V, Mosca A, Miragliotta G (1999). Prevalence of antibodies to *Bartonella henselae* in patients with suspected cat scratch disease (CSD) in Italy. Eur J Epidemiol.

[56] Wass L, Grankvist A, Mattsson M, Gustafsson H, Krogfelt K, Olsen B (2018). Serological reactivity to *Anaplasma phagocytophilum* in neoehrlichiosis patients. Eur J Clin Microbiol Infect Dis.

[57] Curcio SR, Tria LP, Gucwa AL (2016). Seroprevalence of *Babesia microti* in individuals with Lyme disease. Vector Borne Zoonotic Dis.

[58] Duron O, Sidi-Boumedine K, Rousset E, Moutailler S, Jourdain E (2015). The importance of ticks in Q Fever transmission: What has (and has not) been demonstrated?. Trends Parasitol.

[59] Manfredi Selvaggi T, Rezza G, Scagnelli M, Rigoli R, Rassu M, De Lalla F (1996). Investigation of a Q-fever outbreak in northern Italy. Eur J Epidemiol.

[60] Santoro D, Giura R, Colombo MC, Antonelli P, Gramegna M, Gandola O (2004). Q fever in Como, northern Italy. Emerg Infect Dis.

[61] Gern L (2008). *Borrelia burgdorferi* sensu lato, the agent of Lyme borreliosis: life in the wilds. Parasite.

[62] Ortiz de la Tabla V, Berruezo M, García Payá E, Fernández M, García JA, Masiá M (2018). Evaluation of the VirClia^®^ automated chemiluminescent immunoassay system for diagnosing pneumonia caused by *Mycoplasma pneumoniae*. J Clin Lab Anal.

